# A Hierarchical NMPC and TD3-Based Framework for Seamless Cruise-to-Park Automated Valet Parking

**DOI:** 10.3390/s26113409

**Published:** 2026-05-28

**Authors:** Dajie Tian, Levent Guvenc

**Affiliations:** 1 Department of Electrical and Computer Engineering, The Ohio State University, Columbus, OH 43210, USA; tian.952@osu.edu; 2 Automated Driving Lab, Department of Mechanical and Aerospace Engineering, The Ohio State University, Columbus, OH 43212, USA; 3 Department of Mechanical and Aerospace Engineering, The Ohio State University, Columbus, OH 43210, USA

**Keywords:** automated valet parking, NMPC, reinforcement learning, TD3

## Abstract

Automated valet parking requires reliable long-range slot searching and precise low-speed docking in confined structured lots. This paper proposes a hierarchical cruise-to-park framework that combines nonlinear model predictive control (NMPC) for predefined-route cruising with a Twin Delayed Deep Deterministic Policy Gradient (TD3) agent for terminal parking. The system is implemented in a structured Simulink environment with Unreal Engine-based geometry-aware sensing modules. During cruising, a camera-based module detects available slots and triggers the transition to parking. The NMPC uses a custom cost function to improve tracking on curved approaches, while the TD3 policy uses LiDAR feedback and reward shaping with an explicit time penalty to encourage efficient, stable docking. Simulation results demonstrate smooth phase transition, accurate cruising, and effective terminal parking in the training slot. Validation on six previously unseen target slots within the same parking-lot environment shows encouraging intra-lot target-slot transferability without retraining. Additional PPO and SAC comparisons and a time-penalty ablation further evaluate the relative learning performance and the effect of reward design, supporting the proposed architecture as a practical baseline for integrated cruise-to-park automated valet parking studies.

## 1. Introduction

Automated valet parking (AVP) is a key function in intelligent transportation systems because it reduces the burden of low-speed parking, helps prevent minor collisions, and improves the use of limited parking resources in lots and garages [1]. Unlike highway autonomy, AVP operates in confined spaces with frequent curvature changes and tight terminal constraints, requiring both reliable long-range motion execution during cruising and precise low-speed maneuvers into a final target pose during parking. As a result, AVP must address long-horizon tracking and short-horizon high-precision docking simultaneously, while remaining robust to sensing imperfections, model mismatch, and discontinuities caused by curved approaches or mode switching [2,3].

Existing AVP solutions are generally divided into rule-based and learning-based methods [1,4]. Rule-based approaches typically combine geometric or graph-search planners with tracking controllers to achieve predictable behavior and constraint satisfaction in structured maps. Prior studies have shown that such planning and re-planning pipelines can handle narrow lots and perception errors with appropriate sensing configurations [5], and that search-based methods can generate feasible collision-free paths in tight parking scenarios [6]. However, these pipelines often require extensive retuning across layouts and can become fragile when errors accumulate over long approach segments, especially under high curvature, heading wrap-around, or local-minimum effects caused by limitations of low-speed actuation.

Learning-based methods, particularly deep reinforcement learning (DRL), have been explored to reduce hand-crafted rule complexity and to learn policies directly from interaction. Although reinforcement learning has shown promise in autonomous driving, its real-world deployment remains constrained by poor sample efficiency, training instability, and strong dependence on reward design and exploration strategy [4]. These issues are even more pronounced in parking tasks, where sparse rewards provide weak learning signals, slow convergence, and may induce undesirable behaviors such as oscillation or spinning near the goal [4,7]. To alleviate these problems, data-efficient and reward-shaped reinforcement learning formulations have been proposed to improve convergence and better alignment between planning and control objectives [8]. This has motivated hierarchical designs in which model-based and learning-based components handle long-range cruising and precise terminal docking within their respective operating regimes.

Recent AVP research increasingly adopts hierarchical architectures that combine model-based control with reinforcement learning, allowing long-range motion to remain constraint-aware while the final maneuver benefits from learned nonlinear behavior [7]. On the model-based side, optimization-based planners can explicitly enforce kinematic feasibility and collision-avoidance constraints, and have shown strong performance in cluttered or irregular environments, especially when iterative refinement or warm-start strategies are used to maintain practical computation [9,10]. Online re-planning methods have also been studied to preserve trajectory continuity when newly detected obstacles invalidate an ongoing parking plan [11]. In parallel, reinforcement learning has gained attention for low-speed parking because it can capture nonlinear maneuvers with continuous control and has recently demonstrated improved robustness under non-ideal scenarios with modern algorithms [12]. More broadly, MPC-RL integration has been investigated to reduce trial-and-error while preserving constraint structure, such as by using NMPC to assist policy learning or switching, or by learning decision policies that modulate MPC execution [13,14].

Recent studies also indicate that traditional planning methods and hybrid MPC-RL approaches have complementary advantages in cluttered parking environments. Traditional graph-search, geometry-based, and optimization-based planners provide interpretable path generation and explicit constraint handling, which makes them effective for structured lots and obstacle-rich spaces [15,16,17]. However, their performance may depend on map quality, warm-start quality, re-planning frequency, and parameter tuning, especially when the local geometry changes or when perception updates invalidate the planned trajectory. In contrast, hybrid MPC-RL methods attempt to combine the constraint awareness of model-based control with the adaptability of learned policies. Such approaches can use MPC to guide policy learning, assist switching decisions, or maintain feasibility while reinforcement learning improves behavior in nonlinear or difficult-to-model maneuvers [18,19]. Nevertheless, many recent studies still focus on either isolated parking maneuvers, local trajectory planning, or general driving tasks, rather than a complete perception-triggered cruise-to-park AVP process. Therefore, the present work focuses on the integration of NMPC-based cruising, TD3-based terminal parking, and camera-triggered phase switching within one structured parking-lot pipeline, while also evaluating transfer to multiple unseen target slots under fixed controller settings.

Despite this progress, several gaps remain in achieving a seamless cruise-to-park AVP pipeline in a structured parking-lot setting. First, much of the literature still formulates parking as an isolated start-to-slot task, whereas practical AVP requires long-range cruising, perception-driven slot confirmation, and reliable mode switching before the terminal maneuver. Second, long-horizon cruising in confined lots can suffer from tracking error accumulation and discontinuities caused by tight curvature and heading wrap-around, yet these effects are rarely analyzed together with downstream parking performance in an integrated framework. Third, learning-based parking policies often exhibit inefficient near-goal behaviors, such as oscillation or spinning under sparse or delayed rewards, while prior studies do not consistently report stage-wise error trends or demonstrate transfer performance across multiple target slots under fixed controller settings.

Motivated by these gaps, this paper proposes a cruise-to-park AVP framework that couples constraint-aware nonlinear model predictive control (NMPC) for cruising with a learning-based low-speed parking policy. In the implemented system, the vehicle first follows a predefined cruising route in a structured parking lot, and a camera-based free-slot detection module triggers the transition from slot searching to parking once an available space is detected [20]. During cruising and search, the NMPC formulation employs a customized cost function that balances path-tracking accuracy, speed regulation, and control smoothness, enabling accurate long-horizon execution with small tracking errors, including on curved segments. During parking, a Twin Delayed Deep Deterministic Policy Gradient (TD3) agent is trained for continuous control using LiDAR feedback for collision-aware docking [21]. The reward function includes an explicit time-penalty term to suppress pre-parking spinning and to encourage timely convergence to the terminal pose, consistent with the role of reward shaping in improving learning efficiency under sparse or delayed feedback [22]. The overall system is implemented in a structured Simulink environment with Unreal Engine-based geometry-aware sensing modules, enabling consistent evaluation of the perception-triggered phase transition and the end-to-end cruise-to-park process.

The integrated framework is validated through parking-lot simulations in Simulink. Intra-lot target-slot transferability is examined on six previously unseen target slots using identical controller settings and without further retraining, while approach-phase tracking errors and terminal parking errors are recorded for evaluation. The results show stable phase transitions, accurate cruising behavior, and transferable parking performance across the tested slots, suggesting that the proposed NMPC tracker with a customized cost function and the time-penalized TD3 parking policy provide a practical baseline for cruise-to-park AVP studies.

This work does not claim to replace existing hybrid MPC-RL parking frameworks or to demonstrate broad policy generalization across arbitrary parking layouts. Instead, it focuses on an integrated cruise-to-park implementation in which NMPC-based cruising, geometry-based free-slot detection, TD3-based terminal docking, and phase switching are evaluated together under a fixed structured parking-lot setup. The validation therefore emphasizes intra-lot target-slot transferability, baseline algorithm comparison, and reward-design ablation under consistent simulation conditions.

The main contributions of this paper are summarized as follows:

- A hierarchical cruise-to-park AVP framework is developed by integrating camera-triggered slot detection, NMPC-based long-range cruising, and TD3-based low-speed parking within a unified simulation environment.
- A customized NMPC cost design is introduced for the cruising phase to improve long-horizon path-following performance by jointly regulating tracking accuracy, vehicle speed, and control smoothness in structured parking-lot routes.
- A time-penalized TD3 reward formulation is proposed for the parking phase to reduce inefficient oscillation or spinning near the goal and to improve convergence toward the target pose during collision-aware docking.
- The proposed framework is validated on multiple previously unseen parking slots under fixed controller settings to examine phase-transition behavior, docking feasibility, and intra-lot target-slot transferability within the same parking-lot layout. Additional PPO/SAC baseline comparisons and a time-penalty ablation are included to evaluate the relative learning performance and the effect of reward shaping.

The remainder of this paper is organized as follows. Section 2 presents the methodology, including the overall system architecture, the perception-triggered transition logic, the NMPC design for cruising, and the TD3 formulation for parking control. Section 3 reports the simulation settings and experimental results, focusing on cruising performance, terminal parking accuracy, and intra-lot transfer performance across unseen parking slots. Section 4 discusses the main findings, practical implications, and limitations of the proposed framework. Section 5 concludes the paper and suggests directions for future work.

## 2. Methodology

### 2.1. Problem Formulation and System Architecture

This work formulates automated valet parking (AVP) as a cruise-to-park task in a structured parking-lot environment. Starting from an initial pose, the ego vehicle follows a predefined cruising route while searching for an available parking slot, and then performs a low-speed docking maneuver into the target slot under terminal pose constraints. The vehicle pose is defined as $p=[x,y,\psi {]}^{T}$, where x and y denote the planar coordinates in the parking-lot frame and ψ is the yaw angle. The control input is $u=[v,\;\delta {]}^{T}$, where v is the commanded longitudinal speed and δ is the steering command. The control objective is to reach the target pose with bounded terminal errors while satisfying actuation limits and maintaining collision-free motion.

To address the different requirements of long-horizon cruising and short-horizon terminal docking, a hierarchical two-stage control architecture is adopted. During the cruising and slot-search phase, a nonlinear model predictive controller (NMPC) tracks the reference route and regulates vehicle motion under explicit constraints, aiming to reduce tracking error accumulation before entering the terminal parking region. During the final parking phase, a Twin Delayed Deep Deterministic Policy Gradient (TD3)-based controller generates steering actions for precise low-speed docking. In this stage, the longitudinal speed is fixed at 2 m/s, while the TD3 agent determines the steering command.

A phase manager and command selector coordinate the interaction between the two control stages to enable a smooth cruise-to-park transition. During cruising, a camera-based parking-slot perception module monitors slot availability. Once a free slot is confirmed, the perception module provides the corresponding target pose and triggers the switching logic from NMPC cruising to TD3 parking. The phase manager handles the controller handoff, while the command selector routes the selected control commands to the vehicle plant to maintain smooth and well-behaved speed and steering signals. After switching, the TD3 parking controller executes the terminal maneuver using parking-related observations, including relative goal information and LiDAR-derived feedback.

**Figure 1:**
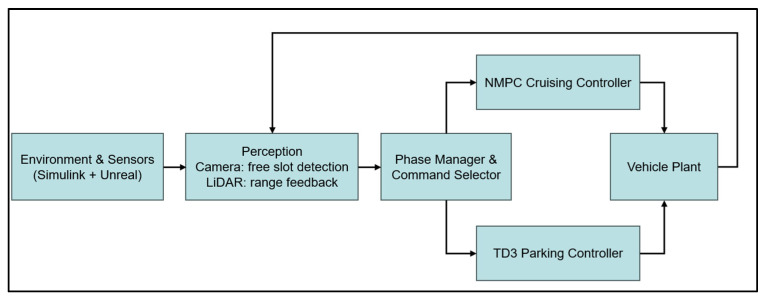
System block diagram of the cruise-to-park AVP framework.

Figure 1 illustrates the overall system architecture, including the simulation environment and vehicle plant, the perception modules, the phase manager and command selector, the NMPC cruising controller, and the TD3 parking controller.

### 2.2. Simulation Environment

All experiments are conducted in MATLAB/Simulink R2025a. The reinforcement learning agent is trained and evaluated using the Reinforcement Learning Toolbox. The closed-loop simulation model integrates the parking-lot environment, the ego-vehicle plant, the perception modules, the phase manager and command selector, the NMPC cruising controller, and the TD3 parking controller. The NMPC module is implemented using MATLAB’s nonlinear MPC functionality in Simulink.

**Figure 2:**
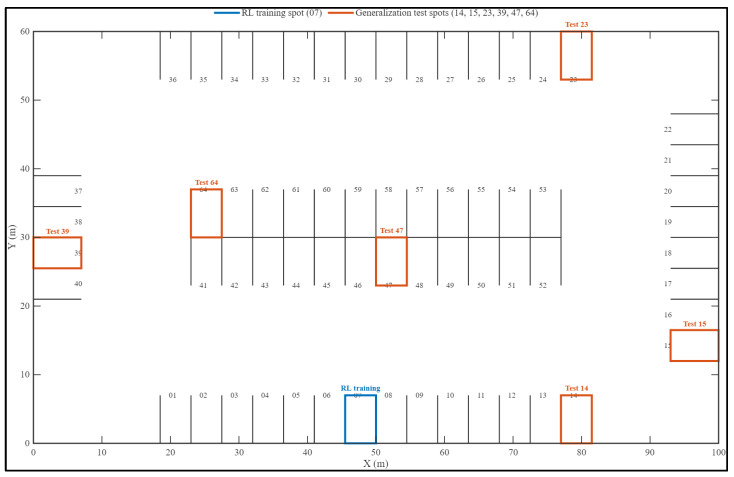
Layout of the parking lot with training spot, marked RL training in blue, and validation test spots, marked Test 14, Test 15, Test 23, Test 39, Test 47 and Test 64 in brown color.

The structured parking-lot layout and slot indexing are adapted from the MathWorks automatic parking valet example [23]. In that baseline, the parking process is organized into a search phase and a parking phase. Building on this layout, the present work implements the proposed NMPC-TD3 cruise-to-park framework, including the controller design, phase-switching logic, and the training and intra-lot transfer evaluation protocol. Figure 2 shows the parking-lot map with indexed parking slots and marked aisles. Each parking slot is assigned a unique index for target selection and experiment organization. In this study, the TD3 policy is trained on a single slot (slot 7) and evaluated on six previously unseen slots (14, 15, 23, 39, 47, and 64) using identical controller settings and without retraining. This indexed layout also enables consistent comparison between training and test scenarios.

All vehicle states and reference quantities are represented in a parking-lot-fixed planar frame, following the pose and control definitions introduced in Section 2.1. To avoid discontinuities in heading-related computations, yaw angles are wrapped to a principal interval using modulo-based mapping, which ensures stable behavior near the ±π boundary. The closed-loop system operates with a discrete controller sample time of $T_{s}=0.1$ s, and both the nonlinear model predictive controller and the TD3 parking controller are executed at this rate. The simulation stop time is set to $T_{f}=50$ s, consistent with the training and evaluation configuration. Sensor outputs are updated at the same effective rate as the controller cycle, enabling synchronized perception and control throughout all experiments.

### 2.3. Vehicle Model and Constraints

**Figure 3:**
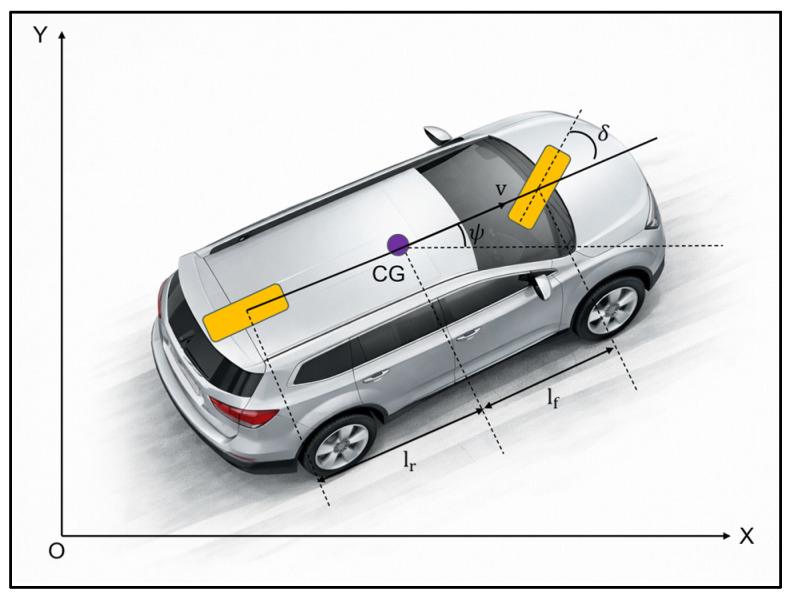
Simplified kinematic bicycle model. The yellow boxes represent the two front wheels and two rear wheels that are lumped together in the single track vehicle model. The purple dot shows the vehicle center of gravity location.

To describe low-speed vehicle motion in the structured parking-lot environment, the ego vehicle is modeled using a planar kinematic bicycle model [24], as shown in Figure 3. The vehicle state is defined by its planar position (X,Y) and yaw angle ψ in the parking-lot-fixed frame, while the control input consists of the longitudinal speed v and front steering angle δ. The vehicle geometry is characterized by the distances from the center of gravity (CG) to the front and rear axles, denoted by $l_{f}$ and $l_{r}$, respectively. In this work, the kinematic bicycle model is used both as the closed-loop vehicle model and as the prediction model for the NMPC cruising controller.

The continuous-time kinematic bicycle model is written as

(1) $$\dot{X}=vcos\psi ,$$

(2) $$\dot{Y}=vsin\psi ,$$

(3) $$\dot{\psi }=\frac{v}{L}tan\delta ,$$

where $L=l_{f}+l_{r}$ is the wheelbase. For digital implementation in Simulink, the continuous-time model is discretized with the controller sampling time $T_{s}$, yielding

(4) $$X_{k+1}=X_{k}+T_{s}v_{k}cos\psi_{k},$$

(5) $$Y_{k+1}=Y_{k}+T_{s}v_{k}sin\psi_{k},$$

(6) $$\psi_{k+1}=\psi_{k}+T_{s}\frac{v_{k}}{L}tan\delta_{k},$$

This discrete-time model is used for closed-loop simulation and NMPC prediction.

To ensure feasible and consistent closed-loop execution, constraints are imposed on both the control inputs and the vehicle motion. The longitudinal speed is bounded by $v\in \left[0,2\right]\;m/s$, restricting the vehicle to forward low-speed operation throughout the maneuver. The steering command is limited to δ∈[−π/4,π/4] rad, consistent with the steering range used during both training and evaluation. In addition, the vehicle motion is constrained within the map-defined workspace limits of the parking lot. Any command exceeding the prescribed bounds is saturated before being applied to the vehicle plant, ensuring that identical actuator limits are enforced for both the NMPC cruising controller and the TD3 parking controller. The timing and actuator constraints used in the experiments are summarized in Table 1.

### 2.4. Perception and Switching Logic

#### 2.4.1. Camera-Based Free Slot Detection

During the cruising stage, a simulated camera module is used to detect an available parking slot ahead of the ego vehicle and to provide a switching trigger for cruise-to-park operation. Unlike pixel-level vision pipelines, this camera is implemented as a geometry-based sensor in the Unreal/Simulink environment. It does not output images, but instead evaluates the visibility of candidate slots using the known parking-lot geometry and returns a binary detection signal together with the target pose of the selected free slot.

Candidate slot locations are represented by a set of predefined slot reference points $\left\{\left(x_{i},y_{i}\right)\right\}$ on the map, where i denotes the slot index. For each slot, the line-of-sight bearing angle and Euclidean distance are computed as

(7) $$\phi_{i}=atan2(y_{i}-y,x_{i}-x),$$

(8) $$d_{i}=\sqrt{{\left(x_{i}-x\right)}^{2}+{\left(y_{i}-y\right)}^{2}},$$

The camera applies forward field-of-view (FoV) and depth gating to determine whether a slot is visible. To ensure robust gating near angle wrap-around boundaries, the bearing can be evaluated either as an absolute line-of-sight angle in the parking-lot frame or as a relative angle with respect to the ego heading. Both representations are equivalent for the FoV test. Using the relative form, the bearing difference is defined as

(9) $${\Delta \phi }_{i}={wrap}_{\pi }(\phi_{i}-\psi ),$$

where ${wrap}_{\pi }(\cdot )$ maps an angle to (−π,π]. A slot is considered visible if

(10) $$\left|{\Delta \phi }_{i}\right|\leq \frac{FoV}{2},$$

(11) $$d_{i}\leq MaxDepth,$$

**Figure 4:**
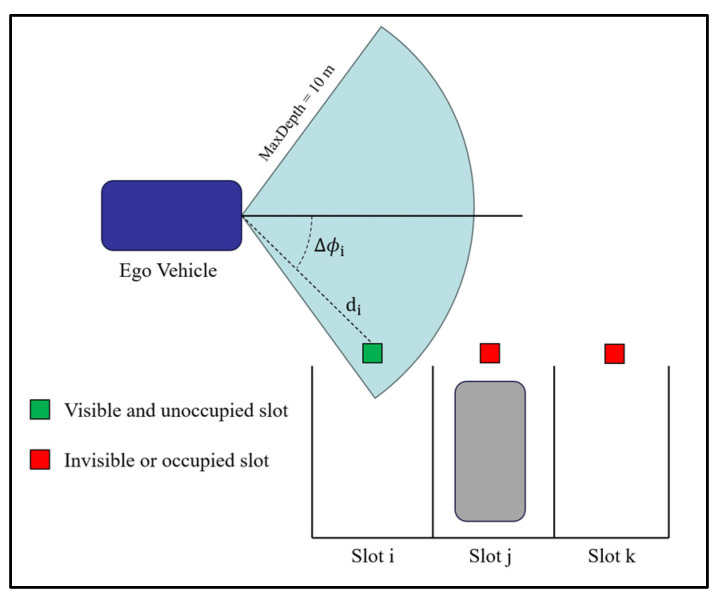
Camera FoV and range gating for free-slot detection. The blue box represents the vehicle. The grey box represents the desired final pose of the parked vehicle. The light green area represents the camera FoV.

Figure 4 illustrates the field-of-view and range-gating geometry used by the camera module. Among the candidate slots that satisfy the FoV and depth constraints and are labeled as unoccupied by the map, an available slot is selected according to the slot indexing order:

(12) $$i^{*}=min\left\{i:\left|{\Delta \phi }_{i}\right|\leq \frac{FoV}{2},d_{i}\leq MaxDepth,\;slot\;i\;is\;unoccupied\right\},$$

The detection signal is set to 1 if the feasible set in (12) is nonempty and 0 otherwise, and the target pose is defined as the docking pose associated with slot $i^{*}$. To prevent intermittent detections from causing oscillatory switching behavior, the target pose is latched once a free slot is detected for the first time, and the latched target pose is held constant thereafter. This latched target pose is then used by the mode-switching logic to trigger the transition from NMPC cruising to TD3 parking. In the reported experiments, MaxDepth=10 m, $FoV=120^{\circ }$, and the camera update rate is synchronized with the controller sampling time of $T_{s}=0.1\;s$.

#### 2.4.2. LiDAR Feedback for Final Parking

During the final parking stage, a simulated LiDAR module provides range-based feedback to support collision-aware docking in tight terminal regions. The LiDAR is implemented as an Unreal/Simulink sensor based on ray casting on the parking-lot geometry. Instead of generating point clouds, it returns a compact range vector that summarizes the nearest obstacle distance along a set of fixed beam directions around the ego vehicle.

Let N denote the number of LiDAR beams. The beam directions are uniformly distributed over $360^{\circ }$ around the ego vehicle, yielding a set of relative beam angles

(13) $$\alpha_{k}=\frac{2\pi (k-1)}{N},\;k=1,\ldots ,N,$$

For each beam k, the LiDAR reports the distance to the nearest obstacle along the corresponding ray direction, forming the range vector

(14) $$d=[d_{1},\;d_{2},\ldots ,\;d_{N}{]}^{⊤}\in R^{N},$$

**Figure 5:**
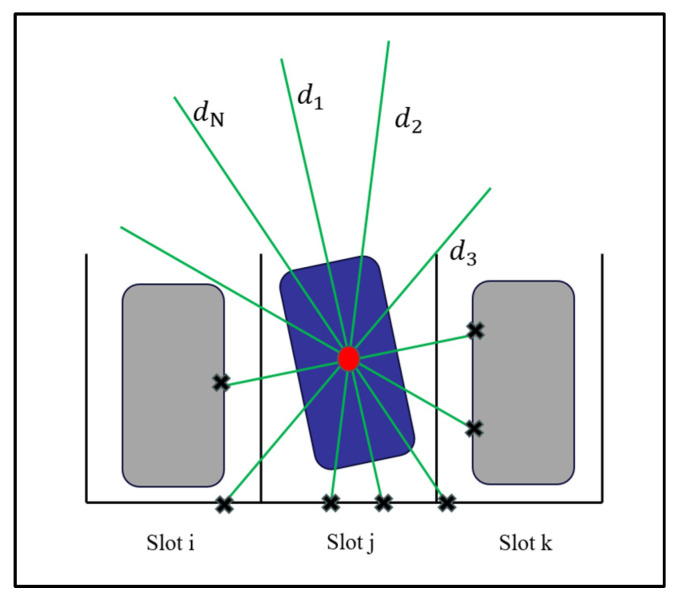
Simulated LiDAR range feedback for final parking. The blue box shows the vehicle during the final parking maneuver. The red circle shows the LiDAR location and the green lines show the LiDAR rays.

Figure 5 illustrates the simulated LiDAR sensing geometry used during final parking. To ensure numerical stability and consistent sensing behavior, range measurements are limited to a valid interval,

(15) $$d_{k}\in \left[d_{min},d_{max}\right],k=1,\ldots ,N,$$

where $d_{max}$ is the maximum sensing range and $d_{min}$ is the minimum valid range. In the reported experiments, N=12 beams are used with $d_{min}=0.5\;m\;$and $d_{max}=6\;m$. The LiDAR update rate is synchronized with the controller sampling time of $T_{s}=0.1\;s$, providing range feedback at each control step during parking.

The LiDAR range vector is embedded directly into the TD3 observation used for the parking policy. Specifically, the observation combines relative goal information, ego motion state, and LiDAR ranges. The LiDAR contributes the N-dimensional vector d as a compact representation of nearby free space and surrounding obstacles. This range-based representation provides an effective perception signal for learning collision-free low-speed parking maneuvers while remaining lightweight and fully synchronized with the closed-loop controller execution.

#### 2.4.3. Seamless Cruise-to-Park Transition

**Figure 6:**
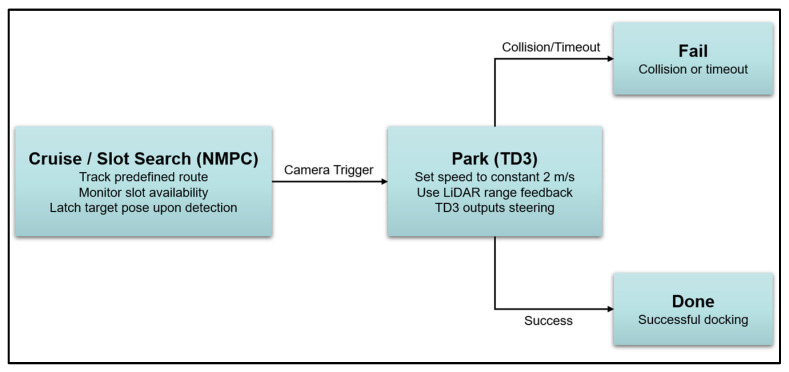
Finite-state machine for seamless cruise-to-park operation.

The integrated phase manager and command selector coordinate the cruising and parking stages to enable a seamless cruise-to-park transition. The switching logic is implemented as a finite-state machine with two operational modes, namely Cruise/Slot Search and Park, followed by two terminal outcomes, Done and Fail, as shown in Figure 6. In the Cruise/Slot Search mode, the NMPC controller tracks the predefined route while the perception modules continuously monitor slot availability. In the Park mode, control authority is transferred to the TD3 parking controller for the terminal maneuver, and the process terminates upon either successful docking or failure caused by collision or timeout.

The transition from Cruise/Slot Search to Park is event-triggered by the camera-based free-slot detection described in Section 2.4.1. Once a free slot is confirmed, the corresponding target pose is latched and used to trigger the switching logic from NMPC cruising to TD3 parking. This event-driven design ensures that the terminal parking phase is activated only after a valid and stable target slot has been identified.

To ensure stable behavior at the switching instant, the integrated phase manager and command selector perform a controlled handover between the two controllers. First, latching the target pose prevents oscillatory switching caused by intermittent detections. Second, consistent actuator limits are enforced across both stages through command saturation, ensuring that the commanded inputs remain within the same admissible bounds before and after the transition. Third, after switching to the park mode, the commanded longitudinal speed is fixed at v=2 m/s, while the TD3 policy provides the steering command for terminal docking. This design yields a stable handover from long-horizon route tracking to short-horizon, collision-aware parking without reinitializing the vehicle state.

### 2.5. NMPC Cruising Controller

#### 2.5.1. NMPC Optimization Formulation

During the Cruise/Slot Search stage, a nonlinear model predictive controller (NMPC) is used to track a predefined reference route in the structured parking lot while respecting actuator limits. At each control update, the NMPC solves a constrained finite-horizon optimization problem using the current vehicle state and a preview of the upcoming reference trajectory [25]. The controller state is defined as $x=[x,y,\psi {]}^{T}$, and the control input is $u=[v,\delta {]}^{T}$, where v is the commanded longitudinal speed and δ is the steering command.

Let p denote the prediction horizon. Given the current state $x_{0}$ and a reference preview ${\left\{r_{k}\right\}}_{k=1}^{p}$ along the cruising route, where $r_{k}=[x_{k}^{ref},\;y_{k}^{ref},\;\psi_{k}^{ref}{]}^{T}$, the NMPC computes an optimal control sequence ${\left\{u_{k}\right\}}_{k=0}^{p-1}$ by minimizing a composite objective that balances route-tracking accuracy, speed regulation, and control smoothness. The optimization problem is formulated as

(16) $${min}_{{\left\{u_{k}\right\}}_{k=0}^{p-1}}\sum_{k=1}^{p}l\left(x_{k},u_{k},\Delta u_{k};r_{k}\right)+l_{T}(x_{p},r_{p}),$$

subject to the discrete-time prediction model

(17) $$x_{k+1}=f\left(x_{k},u_{k}\right),k=0,\ldots ,p-1,$$

and input constraints

(18) $$v_{min}\leq v_{k}\leq v_{max},$$

(19) $$\delta_{min}\leq \delta_{k}\leq \delta_{max},$$

Here, $\Delta u_{k}=u_{k}-u_{k-1}$ denotes the control increment used to encourage smooth actuation. In the implemented controller, the bounds are set to $v_{min}=0\;m/s,\;{\;v}_{max}=2\;m/s,\delta_{min}=-\frac{\pi }{4}\;rad,\;\delta_{max}=\frac{\pi }{4}\;rad$, consistent with the operating constraints defined in Section 2.3.

The NMPC is implemented in Simulink using a nonlinear MPC controller block parameterized by a workspace-defined nonlinear MPC object. At each sampling instant, the finite-horizon optimization problem is solved by a built-in nonlinear programming solver based on sequential quadratic programming [26]. The controller uses the same sampling time as the prediction model, and the reference preview is provided as an external input generated by the route planner as a function of simulation time. To improve numerical stability and reduce step-to-step control discontinuities, the previous control action is fed back to the controller through the dedicated last manipulated variable input with a one-step delay, enabling warm-starting and consistent handling of control increments. The resulting NMPC output is reshaped into a 2×1 column vector to match the downstream command interface $[v,\delta {]}^{T}$. In this work, the controller sampling time is $T_{s}=0.1\;s$, and both the prediction horizon and the control horizon are set to 10 steps.

#### 2.5.2. Custom NMPC Cost Function

A customized NMPC objective is adopted to improve tracking performance on curved cruising segments and reduce error accumulation prior to the cruise-to-park transition. Let the predicted state at step k be $x_{k}=[x_{k},\;y_{k},\;\psi_{k}{]}^{T}$ and the reference preview be $r_{k}=[x_{k}^{ref},\;y_{k}^{ref},\;\psi_{k}^{ref}{]}^{T}$. Define the position differences $\Delta x_{k}=x_{k}-x_{k}^{ref}$ and $\Delta y_{k}=y_{k}-y_{k}^{ref}$. The reference-frame tracking errors are computed as

(20) $$e_{lat,k}=-\sin\left(\psi_{k}^{ref}\right)\Delta x_{k}+\cos\left(\psi_{k}^{ref}\right)\Delta y_{k},$$

(21) $$e_{lon,k}=\cos\left(\psi_{k}^{ref}\right)\Delta x_{k}+sin\left(\psi_{k}^{ref}\right)\Delta y_{k},$$

The heading error is defined with angle wrapping to avoid discontinuities near the ±π boundary:

(22) $$e_{\psi ,k}=wrap\left(\psi_{k}-\psi_{k}^{ref}\right)\in \left(-\pi ,\pi \right],$$

where ${wrap}_{\pi }(\cdot )$ maps an angle to the principal interval (−π,π]. To encourage smooth actuation, control increments are penalized:

(23) $$\Delta v_{k}=v_{k}-v_{k-1},$$

(24) $$\Delta \delta_{k}=\delta_{k}-\delta_{k-1},$$

A terminal error vector is formed from the last-step reference-frame errors:

(25) $$e_{T}=[e_{lat,p},\;e_{lon,p},\;e_{\psi ,p}{]}^{T},$$

Over the prediction horizon p, the customized NMPC cost is defined as

(26) $$J=\sum_{k=1}^{p}(w_{\mathrm{lat}}e_{\mathrm{lat},k}^{2}+w_{\mathrm{lon}}e_{\mathrm{lon},k}^{2}+w_{\psi }e_{\psi ,k}^{2}+w_{v}v_{k}^{2}+w_{\delta }\delta_{k}^{2}+w_{\Delta v}\Delta v_{k}^{2}+w_{\Delta \delta }\Delta \delta_{k}^{2})+w_{T}{∥e_{T}∥}_{2}^{2},$$

This formulation emphasizes reference-frame path tracking through $\left(e_{lat},e_{lon},e_{\psi }\right)$, penalizes excessive control effort and rapid command changes via $\left(v_{k},\delta_{k},\Delta v_{k},\Delta \delta_{k}\right)$, and explicitly penalizes end-of-horizon alignment through the terminal penalty ${∥e_{T}∥}_{2}^{2}$. Using reference-frame errors reduces coupling between global (x,y) deviations and heading changes on curved segments, while the wrapped heading error avoids artificial jumps caused by angle wrap-around. The increment penalties suppress oscillatory behavior that can otherwise amplify tracking errors over long cruising horizons. Since the commanded speed is constrained to be nonnegative in the NMPC formulation, no additional reverse-motion penalty is included in the cost.

The weight values used in this paper are summarized in Table 2. These parameters were selected empirically through trial-and-error tuning in closed-loop simulation. Starting from feasible initial values, the weights were iteratively adjusted to balance reference tracking accuracy, heading alignment, control effort, and control smoothness on representative cruising scenarios, especially curved route segments. The final parameter set reported in Table 2 corresponds to the configuration that provided stable tracking performance with small accumulated error and without excessive oscillation or command chattering. Once selected, the same weight set was fixed for all reported experiments.

A qualitative sensitivity assessment was also conducted during the tuning process to understand the influence of the main weight groups. Increasing the position- and heading-tracking weights generally improved path adherence on curved segments, but excessively large values produced more aggressive steering actions and increased command variation. Conversely, smaller tracking weights allowed smoother inputs but caused larger corner-cutting and greater accumulated lateral error before the parking transition. The terminal penalty was assigned a relatively large value to encourage end-of-horizon alignment with the reference route, which was important for providing a well-conditioned initial pose to the TD3 parking controller. The effort and increment penalties were kept at moderate values so that steering smoothness could be improved without over-damping the controller response in tight-curvature regions. Therefore, the final weights in Table 2 represent a compromise among tracking accuracy, terminal alignment, control smoothness, and actuator feasibility. Once selected, the same values were fixed for all training and validation cases.

### 2.6. TD3 Parking Controller

#### 2.6.1. MDP Formulation

The final parking task is formulated as a continuous-state, continuous-action Markov decision process (MDP), in which the TD3 agent learns a steering policy for low-speed collision-aware docking [27,28]. At each decision step t, the parking environment provides an observation vector $s_{t}$, the agent outputs an action $a_{t}$, and the simulator propagates the vehicle state to the next step according to the closed-loop parking dynamics described in Section 2.3 and Section 2.4.

The observation is a 16-dimensional vector composed of target-relative pose features and LiDAR-based environment feedback. Specifically, the current vehicle pose and the target parking pose are transformed into a target-slot-aligned local frame. Let $e_{x,t}\;$and $e_{y,t}$ denote the relative planar errors with respect to the target pose, and let $e_{\psi ,t}$ denote the relative heading error in the target-aligned frame. To improve numerical conditioning, the position errors are normalized by a factor of 10, while the heading information is represented by its sine and cosine values to avoid discontinuities associated with angular wrap-around. In addition, the LiDAR measurements are normalized by the maximum sensing range $d_{max}$. The state vector is therefore defined as

(27) $$s_{t}={\left[\frac{e_{x,t}}{10},\frac{e_{y,t}}{10},sin(e_{\psi ,t}),cos(e_{\psi ,t}),{\tilde{d}}_{1,t},{\tilde{d}}_{2,t},\ldots ,{\tilde{d}}_{12,t}\right]}^{T}\in R^{16},$$

where ${\tilde{d}}_{i,t}=d_{i,t}/d_{max}$, i=1,…,12, and $d_{max}=6\;m$. In the implemented sensing setup, 12 LiDAR beams are used, consistent with the LiDAR model described in Section 2.4.2.

The action is defined as a one-dimensional continuous steering command,

(28) $$a_{t}=\delta_{t},\delta_{t}\in \left[-\frac{\pi }{4},\frac{\pi }{4}\right],$$

thus, during the parking stage, the TD3 policy controls only the steering angle, while the commanded longitudinal speed is maintained at a constant value of v=2m/s, consistent with the cruise-to-park transition logic described in Section 2.4.3.

An episode terminates under one of three conditions: successful parking, invalid operation, or timeout. Successful parking is declared when the Euclidean position error with respect to the target pose is smaller than 0.75 m and the absolute heading error is smaller than $10^{\circ }$. Invalid operation corresponds to failure cases such as leaving the admissible parking region or approaching an obstacle closer than the minimum valid LiDAR distance threshold, which is set to 0.5 m. During training, an additional invalid condition is triggered when the vehicle heading exceeds 2π, which is used to suppress persistent spinning behavior. A timeout occurs when the episode reaches the maximum allowed duration. In the reported experiments, the controller sampling time is $T_{s}=0.1\;s$, and the maximum episode length is 500 steps, corresponding to a 50 s horizon.

During training, the initial vehicle pose was randomized over several predefined regions of the parking-lot map to improve robustness to different approach conditions. The initial position was sampled either from two fixed entry points or from three subregions spanning the training area, with the yaw angle randomly drawn from the corresponding heading intervals. The initial longitudinal speed was fixed at 2 m/s. This steering-only design was adopted to isolate the low-speed docking policy from longitudinal speed planning and to maintain a consistent parking condition across training, baseline comparison, ablation, and unseen-slot validation. Therefore, the reported TD3 results should be interpreted as steering-policy performance under a fixed low-speed AVP setting rather than a fully coupled longitudinal-lateral parking policy.

#### 2.6.2. Network Architecture and Training Configuration

**Figure 7:**
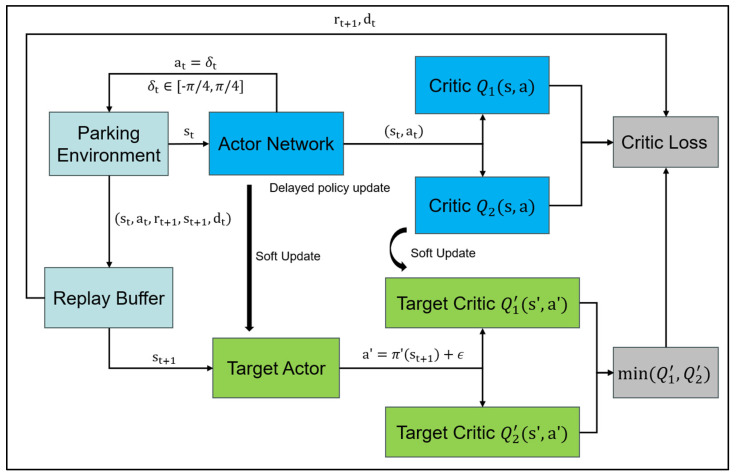
TD3 parking controller framework.

The parking policy is learned using the Twin Delayed Deep Deterministic Policy Gradient (TD3) algorithm [21] displayed in Figure 7. In the implemented framework, the actor network maps the 16-dimensional observation vector to a one-dimensional continuous steering command, while two critic networks estimate the action value for the current state-action pair. Following the TD3 design, the two critics are trained in parallel, the minimum of their target Q-values is used to reduce overestimation bias, the actor is updated less frequently than the critics, and target policy smoothing is applied when forming the bootstrapped target. These mechanisms jointly improve the stability of continuous-control learning.

The actor network takes the normalized parking observation defined in Section 2.6.1 as input. It consists of two fully connected hidden layers with 256 units each, followed by ReLU activations. The output layer is followed by a hyperbolic tangent activation and an output-scaling stage so that the final action remains within the steering bound $\left[-\pi /4,\pi /4\right]$. The critic component adopts the twin-critic structure of TD3. Each critic receives both the observation and the steering action as inputs. In each critic, the state pathway and action pathway are first processed separately and then fused to estimate the scalar Q-value.

During training, transitions $\left(s_{t},a_{t},r_{t+1},s_{t+1}\right)$ are stored in a replay buffer and sampled in mini-batches for off-policy learning. Target networks are maintained for both the actor and the critics, and soft target updates are used after learning iterations. The policy update is delayed with respect to the critic updates, and smoothed target actions are generated by adding clipped noise to the target actor output when computing the critic target. This training setup is consistent with the standard TD3 framework while being specialized to the steering-only parking task considered in this work [21].

The main training hyperparameters used in this work are summarized in Table 3.

Although the maximum training budget was set to 15,000 episodes, an early stopping rule was used in the implemented training process. Specifically, an evaluation statistic was computed every 20 episodes as the mean episode reward over the evaluation window, and training was terminated once this value exceeded 140. Under this criterion, the reported parking policy converged and stopped after 850 episodes.

#### 2.6.3. Reward Shaping with an Explicit Time Penalty

To improve parking efficiency and suppress oscillatory behavior near the target pose, the TD3 parking policy is trained with a shaped reward that combines distance-based incentives, orientation alignment, control regularization, terminal bonuses and penalties, and an explicit per-step time penalty [29,30]. The total reward at step t is defined as

(29) $$r_{t}=r_{dist,t}+r_{prog,t}+r_{ori,t}+r_{ctrl,t}+r_{park,t}+r_{invalid,t}+r_{time,t},$$

The distance reward encourages the vehicle to approach the target parking pose in the planar position space. Let $e_{x,t}$ and $e_{y,t}$ denote the unnormalized target-relative position errors. In the implementation, these quantities are recovered from the normalized observation entries by multiplying the first two state components by 10. The distance reward is defined as

(30) $$r_{dist,t}=2exp(-0.05{e_{x,t}}^{2}-0.04{e_{y,t}}^{2}),$$

To additionally reward step-to-step improvement toward the goal, a progress reward is introduced using the increase in the distance reward relative to the previous control step. The reward increment is clipped to the interval [0,0.1] before scaling, yielding

(31) $$r_{prog,t}=3{sat}_{\left[0,0.1\right]}(r_{dist,t}-r_{dist,t-1}),$$

where ${sat}_{\left[0,0.1\right]}(\cdot )$ denotes saturation to the interval [0,0.1]. This design rewards positive progress while preventing excessively large stepwise reward jumps.

The orientation reward promotes heading alignment with the target parking pose. Let $e_{\psi ,t}=\psi_{t}-\psi_{g}$ denote the heading mismatch between the current and target poses. The orientation term is defined as

(32) $$r_{ori,t}=0.1exp(-20{e_{\psi ,t}}^{2}),$$

To discourage unnecessarily large steering commands and rapid steering oscillations, a control penalty is applied to both the steering action and its step-to-step variation:

(33) $$r_{ctrl,t}=-0.05{\delta_{t}}^{2}-0.1{\left(\delta_{t}-\delta_{t-1}\right)}^{2},$$

In addition to these shaping terms, terminal rewards are used to distinguish successful parking and invalid outcomes. A successful parking event produces a positive terminal bonus,

(34) $$r_{park,t}=100I_{parked,t},$$

while invalid operation, including leaving the admissible region or approaching obstacles closer than the minimum LiDAR safety threshold, incurs a penalty,

(35) $$r_{invalid,t}=-50I_{invalid,t},$$

where $I_{parked,t}\;$and $I_{invalid,t}$ are binary indicators for successful parking and invalid termination, respectively.

To explicitly discourage lingering, repeated spinning, or oscillatory maneuvers that do not lead to task completion, a per-step time penalty is applied whenever the episode is still active:

(36) $$r_{time,t}=-0.02(1-I_{parked,t})(1-I_{invalid,t}),$$

This term contributes a constant negative reward at every nonterminal step. As a result, behaviors that waste time without improving the parking state accumulate additional penalty. In particular, spinning or oscillatory corrections near the target become less favorable unless they lead to measurable progress or immediate task completion. The explicit time penalty therefore biases the learned policy toward shorter and more decisive parking maneuvers, reducing time-to-park while complementing the distance, orientation, and progress rewards [30].

The reward coefficients used in this work are summarized in Table 4.

The reward coefficients in Table 4 were selected to balance terminal accuracy, learning stability, maneuver efficiency, and collision avoidance. The distance and progress terms provide dense feedback during the approach to the target pose, which helps avoid the sparse-reward problem commonly observed in parking tasks. The longitudinal and lateral position weights were chosen with comparable magnitudes so that the policy would not overfit to only one direction of terminal error. The orientation term was assigned a relatively strong exponential penalty because correct heading alignment is essential for successful docking into a narrow parking slot. The steering and steering-increment penalties were kept smaller than the goal-related rewards so that the agent could still generate sufficiently large steering commands when needed, while avoiding unnecessary oscillations. The parking bonus and invalid-operation penalty were selected to clearly separate successful and unsafe terminal outcomes. The time penalty was intentionally set to a small per-step value. Over a long episode, it becomes large enough to discourage lingering and spinning, but it is not so large that it dominates the distance, orientation, and safety-related terms. During pilot training, a weaker time penalty led to longer near-goal oscillations, while an excessively strong time penalty encouraged premature or overly aggressive maneuvers. The final coefficient set was therefore retained as a balanced configuration and was kept unchanged in all reported experiments.

### 2.7. Metrics and Evaluation Setup

#### 2.7.1. Training–Validation Split and Intra-Lot Transfer Setup

To evaluate the intra-lot target-slot transfer capability of the proposed cruise-to-park framework, the parking policy is trained on a single target slot and then validated on multiple previously unseen target slots without retraining. In the reported implementation, the TD3 parking controller is trained using slot 7 as the only training target, while the NMPC cruising controller, perception modules, and phase-switching logic remain fixed throughout both training and validation.

The single-slot training setup is adopted intentionally to examine whether the learned low-speed parking policy can capture transferable terminal maneuvering behavior within the same structured parking-lot layout rather than memorizing only one slot-specific trajectory. Slot 7 is selected as the training target because it provides a representative parking configuration within the structured lot while allowing repeated closed-loop interaction under consistent sensing, actuation, and transition conditions. By restricting training to one slot, the subsequent validation can more clearly evaluate the intra-lot target-slot transfer performance of the learned parking controller across different target locations within the same structured parking-lot layout.

After training, the learned policy is validated on six previously unseen target slots, namely slots 14, 15, 23, 39, 47, and 64. During this evaluation stage, all controller settings are kept unchanged, including the NMPC configuration, the TD3 network parameters, the reward function, the parking success criteria, and the perception-triggered switching logic. No additional fine-tuning, retraining, or slot-specific parameter adjustment is performed. Therefore, performance differences across the validation slots reflect the intra-lot target-slot transfer behavior of the learned parking policy under fixed controller settings, rather than slot-specific adaptation or online fine-tuning for each individual target.

This train-on-one-slot and validate-on-multiple-unseen-slots protocol is used to assess whether the proposed hierarchical framework can maintain reliable cruise-to-park execution when the terminal parking target changes within the same parking-lot environment. Therefore, the validation should be interpreted as intra-lot target-slot transfer under a fixed map and fixed controller design, rather than broad policy generalization across different parking-lot layouts, aisle widths, sensing conditions, or dynamic traffic scenarios.

#### 2.7.2. Metrics and Logging

To evaluate the performance of the proposed framework, both cruising-stage tracking quality and terminal parking accuracy were recorded during closed-loop simulation. Since the overall system follows a hierarchical cruise-to-park structure, the logged metrics are organized according to the two operational stages and the controller handoff between them.

For the Cruise/Slot Search stage, the main evaluation quantities were the lateral tracking error and heading error defined in the reference frame of the cruising route, as introduced in Section 2.5.2. These quantities were used to assess how accurately the NMPC controller followed the predefined route before the parking transition was triggered. In addition to the full-time histories of the tracking errors, the approach-state error at the switching instant was also recorded to characterize the vehicle state passing from NMPC cruising to TD3 parking.

To evaluate whether the controller handoff induces transient instability, additional switching-related quantities are logged at the cruise-to-park transition. These include the lateral error, heading error, steering command before and after switching, steering-command jump, speed command before and after switching, and whether repeated mode switching occurs. These metrics are used to verify that the target-pose latching and command-selection logic produce a stable transition from the NMPC controller to the TD3 parking controller.

For the final parking stage, the main metrics were the terminal position error, terminal heading error, parking outcome, and time-to-park. The terminal position error was computed as the Euclidean distance between the final vehicle position and the target parking pose, while the terminal heading error was defined as the absolute yaw difference with respect to the target orientation. The time-to-park was measured over the parking phase after the cruise-to-park transition was activated. Invalid termination events, including collision-related failure or leaving the admissible region, were also logged.

In addition to terminal pose error, the parking success rate and final footprint containment are evaluated. The success rate is computed as the ratio between the number of successful parking trials and the total number of evaluated target-slot trials. A trial is considered successful if the vehicle reaches the terminal parking condition without collision, invalid termination, or timeout. To further examine whether the final pose is physically contained within the target parking slot, the four vehicle corner points are computed from the final vehicle pose and vehicle dimensions, and a binary final-footprint containment indicator is recorded for each target slot.

To provide a preliminary assessment of computational feasibility, the NMPC optimization solve time is also logged during the cruising phase. The average NMPC solve time per control step is computed and compared with the controller sampling time of 0.1 s. This metric is used to assess whether the current MATLAB/Simulink implementation is compatible with the selected simulation control rate, while recognizing that embedded deployment would require additional code generation and hardware-specific profiling.

In the reported experiments, the results are presented as slot-wise closed-loop evaluation outcomes under fixed controller settings. For each target slot, the recorded metrics include parking duration, terminal position error, lateral and longitudinal terminal errors, and terminal heading error. Aggregate mean values across the six unseen-slot tests are additionally reported to summarize overall transfer performance. This evaluation protocol enables both stage-specific analysis and overall assessment of the end-to-end cruise-to-park behavior under a consistent testing configuration.

All evaluations were conducted using the same trained TD3 policy and the same fixed NMPC, perception, and switching parameters described in the methodology section. No retraining or slot-specific retuning was introduced during testing, so the reported metrics directly reflect the transferability of the integrated framework under a fixed parking-lot setup.

It should be noted that the reported slot-wise evaluation was conducted in a deterministic Simulink closed-loop environment. For a given target slot, the trained TD3 policy, NMPC controller, perception-triggered switching logic, actuator constraints, and initial test configuration were all kept fixed. In addition, the target pose was latched once the free slot was detected, which ensured a consistent cruise-to-park transition point for the same test case. Under this deterministic setup, repeated simulations of the same slot produced identical closed-loop outcomes. Therefore, the reported result for each slot is representative of that fixed evaluation condition rather than a stochastic average over multiple randomized trials.

#### 2.7.3. Baseline Comparison and Reward Ablation Protocol

To provide quantitative comparisons against alternative reinforcement learning approaches, additional PPO and SAC agents are trained and evaluated under the same parking environment. The PPO, TD3, and SAC agents use the same observation definition, action range, controller sampling time, terminal success criteria, invalid termination conditions, and training target slot. The comparison is designed to assess learning convergence, parking feasibility, terminal accuracy, and maneuver efficiency under consistent simulation conditions, rather than to establish a universal ranking among reinforcement learning algorithms.

For training convergence comparison, the episode reward and the 20-episode moving mean reward are recorded for each algorithm. The same evaluation window and stopping threshold are used where applicable so that convergence behavior can be compared under a consistent criterion. For terminal parking comparison, the trained policies are evaluated using parking duration, final position error, final heading error, and parking success outcome.

To directly examine the effect of the explicit time-penalty term in the TD3 reward function, a reward ablation study is conducted. In this ablation, the TD3 agent is retrained after removing the per-step time-penalty term, while the remaining reward components, observation definition, action range, training target, and termination conditions are kept unchanged. The resulting policy is compared with the proposed time-penalized TD3 policy in terms of training convergence behavior, terminal parking feasibility, and qualitative near-goal motion. This comparison focuses on whether removing the time penalty leads to longer lingering, repeated corrective motion, or spinning-like behavior near the target region, rather than providing a full component-wise reward decomposition.

## 3. Results

### 3.1. Overall Performance of the Proposed AVP Framework

**Figure 8:**
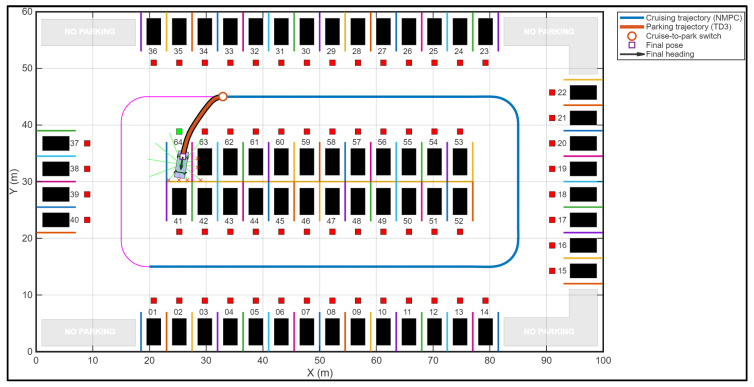
Representative cruise-to-park trajectory for slot 64.

To evaluate the end-to-end capability of the proposed AVP framework, a representative cruise-to-park trial for slot 64 was examined in the structured parking-lot environment. As shown in Figure 8, the ego vehicle successfully completed the full task from the initial position to the target slot. The vehicle first followed the predefined cruising route under the NMPC cruising controller and then switched to the TD3 parking controller for the terminal docking maneuver after the target slot was confirmed by the perception module. The task was completed successfully without collision or invalid termination. Slot 64 was selected as the representative end-to-end case because it illustrates the complete cruise-to-park process in one of the unseen target-slot scenarios.

The full trajectory demonstrates that the proposed framework successfully integrates long-range constrained cruising and low-speed parking within a unified closed-loop architecture. During the cruising phase, the NMPC controller maintained stable path-following behavior along the predefined route and guided the vehicle smoothly toward the parking region. After the cruise-to-park switching point, the TD3 controller generated the steering actions required for precise low-speed docking into slot 64. No obvious trajectory discontinuity was observed around the controller handoff, indicating that the phase manager and command selector achieved the intended smooth transition between the two control stages.

Overall, this representative result confirms the feasibility of the proposed cruise-to-park AVP pipeline in a structured parking-lot scenario. The successful completion of the full maneuver indicates that the perception-triggered switching logic, the NMPC cruising controller, and the TD3 parking controller can operate coherently within the proposed end-to-end AVP framework.

### 3.2. NMPC Cruising Results

The cruising-stage tracking performance of the proposed framework was evaluated using the representative test for slot 64. During the cruising stage, the NMPC controller guided the vehicle along the predefined route before the parking maneuver was triggered. The lateral tracking error and heading error remained bounded throughout the approach phase, indicating that the vehicle entered the parking stage with a well-aligned pose for the subsequent TD3 parking controller.

**Figure 9:**
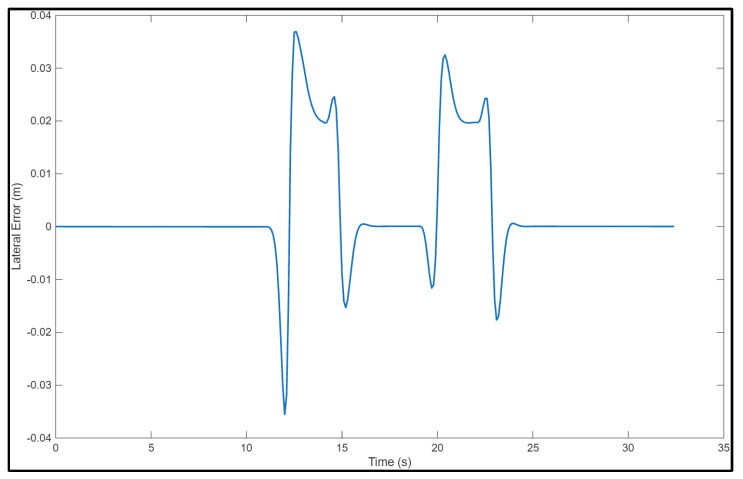
Lateral tracking error during NMPC cruising for slot 64.

Figure 9 shows the lateral tracking error during the NMPC cruising stage. Since the cruising controller primarily aims to keep the vehicle aligned with the reference path, the evaluation focuses on lateral deviation and heading alignment rather than longitudinal tracking error. The lateral error remained bounded within a small range throughout the approach phase, with larger deviations mainly appearing during curved segments and local geometric transitions of the route. Quantitatively, the cruising-stage tracking remained accurate, with a maximum lateral deviation of 0.0369 m and a mean absolute lateral error of 0.0051 m. These transient peaks were limited in magnitude and decayed quickly after the vehicle completed the corresponding turning adjustments, demonstrating effective path regulation under the customized NMPC formulation.

**Figure 10:**
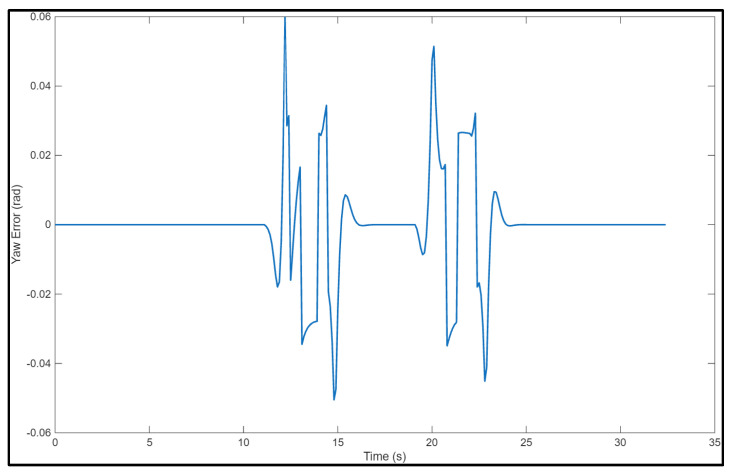
Heading error during NMPC cruising for slot 64.

Figure 10 presents the heading error of the vehicle relative to the local path tangent during cruising. The heading error remained well controlled overall and was mainly concentrated around the same turning regions where lateral correction was required. Although several short-duration peaks appeared during curvature changes, the error quickly returned toward zero after each transition, indicating that the controller regulated the vehicle orientation without persistent oscillation. The final yaw error at the end of the cruising phase was approximately zero, further confirming that the vehicle entered the parking stage with a well-aligned pose.

Overall, the NMPC controller achieved accurate and well-behaved cruising motion before the phase switch to parking. The bounded lateral deviation and rapidly recovering heading response indicate that the controller provided a stable pre-parking trajectory, which is important for ensuring a smooth handoff to the TD3 parking controller in the integrated AVP framework.

The cruise-to-park switching behavior was further examined from the closed-loop trajectory and phase-transition response. Once a valid parking slot was detected, the target pose was latched and the system switched from NMPC cruising to TD3 parking without repeated mode switching. No visible trajectory discontinuity, collision, workspace violation, or post-switch oscillatory behavior was observed around the handoff point in the reported simulations. This indicates that the proposed latching and command-selection logic provided a smooth controller handoff under the deterministic simulation conditions considered in this study.

The NMPC solve time was estimated using the Simulink Profiler during the cruising phase. Since the NMPC optimization is implemented inside the MPC Tracking Controller block, the profiled execution time of this block was used as a conservative implementation-level estimate of the NMPC solve time. The profiled MPC Tracking Controller required an average of 0.0405 s per controller call, calculated from a total profiled time of 32.897 s over 813 calls. This value is below the controller sampling time of 0.1 s, suggesting that the current MATLAB/Simulink implementation is preliminarily feasible for the selected simulation control rate. Nevertheless, the reported value should be interpreted as a profiler-based controller-level timing estimate rather than a certified embedded real-time solver benchmark, and embedded deployment would require code generation, hardware-specific profiling, and validation under processor and communication constraints.

### 3.3. TD3 Parking Results

The parking-stage performance of the proposed framework was evaluated using the TD3 training process and the terminal parking behavior in the training slot. Since the parking policy was trained in slot 7, this section focuses on the learned parking behavior in that slot before the unseen-slot transfer results are presented in Section 3.4.

**Figure 11:**
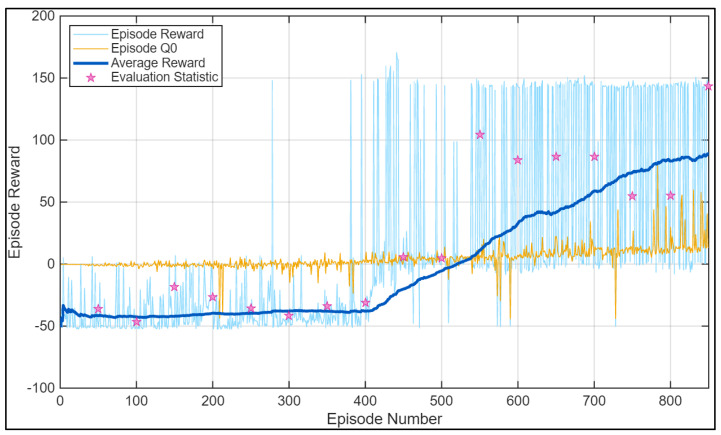
TD3 training reward curve for parking policy learning in slot 7.

Figure 11 shows the TD3 training reward curve for parking policy learning. As training progressed, the episode reward exhibited an overall increasing trend, while the average reward and evaluation statistics also improved gradually. Training was terminated after 850 episodes once the stopping criterion was satisfied. In the implemented training setup, the evaluation statistic was computed every 20 episodes as the mean episode reward over the evaluation window, and training stopped when this value exceeded 140. At termination, the final average reward was 89.0592 and the evaluation statistic reached 143.201. These results indicate that the agent progressively learned more effective parking behaviors during training. The stabilization of the reward trend in the later training stage suggests that the proposed reward formulation provided informative learning signals and supported convergence of the parking policy.

**Figure 12:**
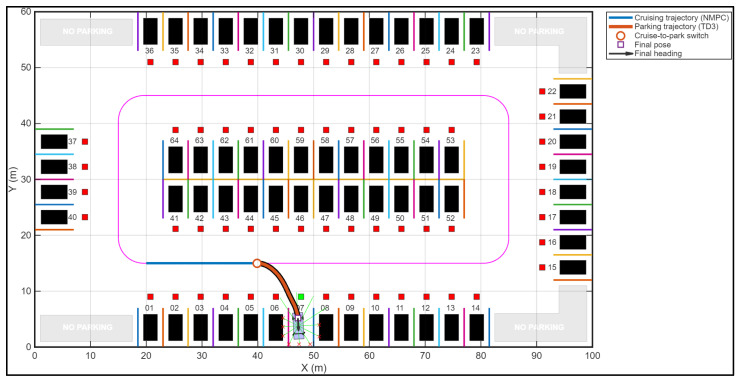
Parking trajectory and final pose in training slot 7.

The learned policy was further examined in the training slot to evaluate its terminal docking performance. As shown in Figure 12, the vehicle successfully executed the parking maneuver in slot 7 and reached the target parking region with stable low-speed behavior. The terminal trajectory remained smooth, and no obvious oscillatory correction or unstable spinning behavior was observed near the goal, indicating that the TD3 controller was able to generate effective steering actions for the final docking stage.

Quantitatively, the parking maneuver in slot 7 was completed in 6.50 s. The final position error was 0.6270 m, including a lateral error of −0.5615 m and a longitudinal error of −0.2791 m, while the final yaw error was 0.0534 rad (3.06 deg). In addition, the trajectory-wide RMS distance-to-goal during parking was 7.8054 m and the maximum distance-to-goal was 12.7944 m, reflecting the large initial offset before convergence to the target pose. These results indicate that the learned policy achieved successful and stable terminal parking in the training slot, with good final heading alignment and reasonable terminal accuracy.

Overall, the TD3 training and parking results confirm that the learned parking policy can provide a reliable low-speed docking controller within the proposed cruise-to-park AVP framework. Together with the designed reward shaping strategy, the controller enables stable parking behavior in the trained scenario and establishes the basis for the unseen-slot validation study presented in the next section.

### 3.4. Validation in Unseen Slots

The intra-lot target-slot transferability of the learned parking policy was validated on six previously unseen parking slots, namely Slots 14, 15, 23, 39, 47, and 64. All tests were conducted using the same controller settings as in the training slot, and no additional retraining or parameter adjustment was performed. This evaluation was intended to examine whether the learned TD3 parking policy could be transferred to new terminal parking scenarios within the same structured parking-lot environment.

**Figure 13:**
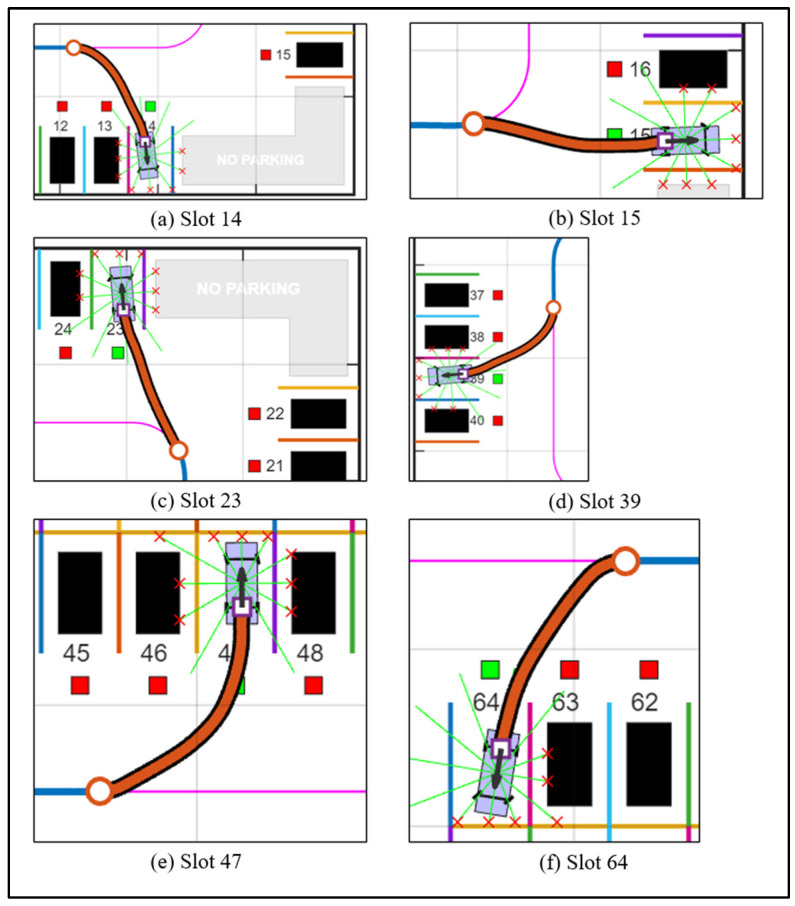
Parking trajectories and final poses in unseen slots 14, 15, 23, 39, 47, and 64.

As summarized in Table 5, the learned policy completed successful docking maneuvers in all six unseen validation slots under the same fixed controller settings. No collision, invalid termination, workspace violation, or timeout was observed in these tests, corresponding to a success rate of 100% under the deterministic evaluation setting. Since the vehicle is expected to approach the target slot from outside during the terminal maneuver, parking-slot containment was evaluated at the final pose rather than imposed as an in-slot constraint throughout the entire approach. During the reported validation trials, no workspace-boundary violation, obstacle collision, invalid termination, or final footprint violation was observed. The computed final-footprint containment indicator was true for all six unseen-slot validation cases. The final-pose visualization in Figure 13 further confirms that all final vehicle footprints were contained within their corresponding target parking regions. The parking duration ranged from 6.4 s to 7.4 s. The final position error remained below 0.75 m in Slots 14, 15, 23, and 39, with corresponding final yaw errors of 0.1164 rad, 0.0362 rad, 0.0803 rad, and 0.0880 rad, respectively. More challenging but still successful cases were observed in Slots 47 and 64, where the final position errors increased to 1.3380 m and 1.1849 m, respectively. These two cases did not correspond to parking failure. Instead, the controller still guided the vehicle into the designated parking region with stable terminal behavior and acceptable final orientation, while exhibiting larger terminal offsets than the other validation-slot tests. This indicates that the main limitation in these cases was reduced terminal accuracy rather than loss of parking feasibility. Over all six validation-slot tests, the mean final position error was 0.8748 m and the mean final yaw error was 0.0818 rad (4.69 deg), indicating that the learned policy retained encouraging intra-lot target-slot transfer performance for the validation parking conditions that were not part of the training.

The larger terminal position errors observed in Slots 47 and 64 can be attributed to the greater mismatch between their local approach geometries and the training-slot condition. In these two cases, the vehicle enters the parking phase from a relative pose that is less similar to the training distribution associated with Slot 7. As a result, the TD3 controller must correct a larger terminal offset within a short low-speed docking horizon. The component-wise errors in Table 5 also indicate that the main degradation is positional rather than purely orientational. For example, Slot 47 has a very small final yaw error but a larger lateral and longitudinal offset, suggesting that the vehicle remained well aligned in heading while stopping at a shifted terminal position. Slot 64 also shows a larger combined positional offset, although the vehicle still reached the designated parking region without invalid termination. These results suggest that the learned policy achieves intra-lot target-slot transferability, but it is not fully invariant to slot-specific approach geometry when trained on only one target slot. Future work can mitigate this limitation by using multi-slot training, domain randomization over target poses and approach angles, slot-geometry-aware observation features, or online fine-tuning and adaptation for new parking layouts.

Figure 13 shows the local parking trajectories and final poses in the six validation slots. Although the target locations and approach geometries differed across cases, the learned TD3 controller generated feasible docking trajectories without retraining. The larger terminal offsets in Slots 47 and 64 suggest that terminal accuracy remains sensitive to local approach geometry, but these cases still achieved stable docking within the designated parking region. Overall, the validation results indicate that the policy learned transferable parking behavior within the structured parking-lot environment rather than memorizing the training slot.

### 3.5. Baseline Comparison and Reward Ablation

#### 3.5.1. Baseline Comparison Among TD3, PPO, and SAC

To provide a quantitative comparison against alternative reinforcement learning baselines, PPO and SAC agents were additionally trained and evaluated under the same parking environment. The three algorithms used the same observation definition, action space, reward formulation, training target slot, and six-slot evaluation protocol. The comparison is intended to provide an algorithmic baseline under the implemented simulation setting rather than to establish a universal ranking of reinforcement learning methods for all AVP scenarios.

Table 6 summarizes the cross-algorithm comparison. PPO showed the fastest training convergence and better sample efficiency, while SAC showed stronger exploration ability but less stable terminal behavior in the more challenging validation cases. In terms of parking outcomes, both TD3 and PPO completed all six validation slots, whereas SAC produced an unstable trajectory with a large terminal error in Slot 64. The performance gap was most evident in the difficult cases, as reflected by the worst-case error, where TD3 achieved the lowest worst-case position error among the three algorithms. Therefore, TD3 was retained as the main parking controller in the proposed cruise-to-park framework because it provided the most reliable balance between terminal accuracy, stability, and robustness across the six-slot evaluation.

The comparison indicates that PPO had the best training efficiency, while TD3 provided the most reliable terminal parking performance across the six validation slots. SAC showed competitive behavior in easier slots, but its unstable looping behavior and large terminal error in Slot 64 reduced its overall reliability.

#### 3.5.2. Ablation Study on the Explicit Time Penalty

To directly examine the effect of the explicit time-penalty term in the TD3 reward function, an ablation study was conducted by retraining the TD3 agent after removing the per-step time penalty, while keeping the observation definition, action space, remaining reward components, training target slot, and termination conditions unchanged. This experiment was designed to evaluate whether the explicit time penalty indeed helps suppress inefficient near-goal behavior during terminal docking.

**Figure 14:**
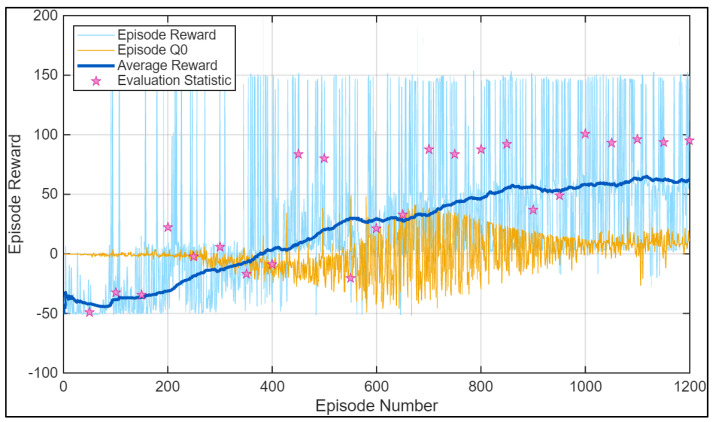
Training reward evolution of TD3 without the explicit time penalty.

Figure 14 shows the training reward evolution of the TD3 agent without the explicit time penalty. Relative to the time-penalized TD3 training results reported in the previous experiments, the ablated case exhibited a weaker reward-improvement trend and less stable convergence behavior. This suggests that the explicit time penalty contributes to more efficient policy learning by discouraging unproductive near-goal motion, rather than merely changing the final parking pose.

**Figure 15:**
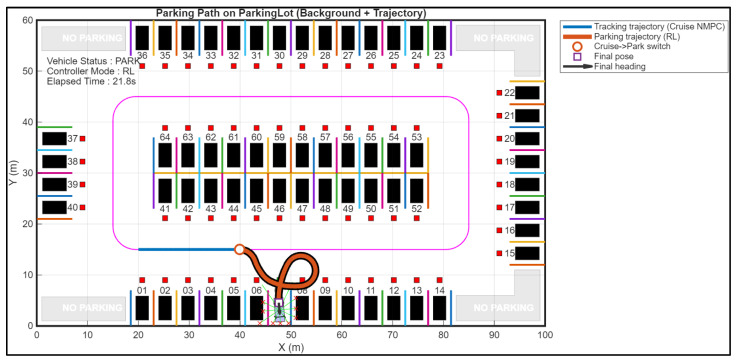
Parking path of the ablated TD3 policy without the explicit time penalty.

Figure 15 presents a representative parking trajectory obtained without the explicit time penalty. In this case, the vehicle exhibited noticeable spinning-like and repeated corrective motion near the target region before final docking. Although the agent was still able to enter the designated parking region, the terminal maneuver became less decisive and required more unnecessary adjustment, indicating that parking feasibility was largely preserved while parking efficiency deteriorated. Compared with the proposed reward design, the ablated policy showed more prolonged near-goal corrective motion, indicating reduced maneuver decisiveness in this representative case.

Overall, the ablation results support the inclusion of the explicit time penalty in the proposed reward formulation. The main benefit of this term is to suppress spinning-like and inefficient near-goal motion, improve training efficiency, and promote smoother and more decisive terminal parking behavior.

However, the present ablation study was intentionally focused on the explicit time-penalty term, because this term was directly associated with the spinning-like and inefficient near-goal behavior observed during pilot training. The progress reward and steering-smoothness penalty were kept unchanged in order to isolate the contribution of the time penalty. A more comprehensive component-wise reward ablation, including separate evaluations of the progress reward and the steering-smoothness penalty, will be conducted in future work.

## 4. Discussion

The results support the effectiveness of separating the AVP task into long-horizon cruising and terminal parking. In this structure, NMPC provides a constraint-aware tracking layer before the switching point, while TD3 focuses on the nonlinear low-speed maneuver near the target slot. This division reduces the burden on a single controller and allows each component to operate within a more suitable regime. The observed cruise-to-park behavior suggests that such a hierarchical design can provide a practical baseline for integrated AVP studies in structured parking-lot environments [31]. The profiled MPC tracking controller block required an average of 0.0405 s per controller call, which was below the 0.1 s controller sampling time. This result provides a preliminary indication of computational feasibility under the selected MATLAB/Simulink simulation setting, although embedded real-time deployment would still require code generation and hardware-specific profiling.

The baseline comparison further supports the selection of TD3 as the main parking controller in the proposed framework. PPO showed the fastest training convergence, indicating higher training efficiency under the implemented reward and observation settings. SAC maintained stronger exploration ability, but its terminal behavior became less stable in the more challenging validation cases, especially in Slot 64. In contrast, TD3 achieved the lowest worst-case position error and the most reliable terminal behavior across the six-slot evaluation. Therefore, the comparison does not imply that TD3 is universally superior to PPO or SAC, but it shows that TD3 provided the best balance among terminal accuracy, stability, and robustness in the implemented parking environment.

The reward design also played an important role in shaping the parking behavior. The ablation study showed that removing the explicit time-penalty term weakened the training trend and produced spinning-like or repeated corrective motion near the target region. This directly supports the role of the time penalty in discouraging inefficient near-goal behavior. In the proposed time-penalized TD3 setting, no obvious repeated spinning behavior was observed near the goal, suggesting that the time-penalized reward helped suppress locally inefficient behaviors while preserving stable convergence. This finding is consistent with prior studies showing that reinforcement learning-based parking performance is highly sensitive to reward formulation and task structure [29,30,32,33]. Nevertheless, the current ablation does not fully decompose all reward components. Future experiments will further examine the independent and coupled effects of the progress reward, steering magnitude penalty, and steering-increment penalty on convergence speed, trajectory smoothness, time-to-park, and terminal parking accuracy.

The validation results further suggest that the learned policy did not merely memorize the training slot. Under fixed controller settings and without retraining, the controller completed successful docking maneuvers in all six unseen validation slots within the same structured parking-lot layout. However, this result should be interpreted as intra-lot target-slot transferability rather than broad policy generalization across different parking environments. The variation in terminal position error, especially in Slots 47 and 64, indicates that parking accuracy remains sensitive to target-slot geometry and relative pose at the switching point. This suggests that the learned docking strategy captured transferable behavior within the tested structured parking lot, while further improvements are still needed to reduce slot-dependent terminal offsets.

### 4.1. Modeling Assumptions and Practical Limitations

The present study is based on several modeling assumptions that are appropriate for an initial cruise-to-park validation but should be considered when interpreting the results. First, the kinematic bicycle model is suitable for the low-speed AVP setting considered in this work, where tire slip and high-order lateral dynamics are less dominant than in high-speed maneuvers [34]. However, this model does not explicitly capture tire saturation, suspension effects, actuator dynamics, or low-friction conditions. These effects may influence tracking accuracy and phase-transition quality in real vehicles.

Second, the camera-based free-slot detection module is implemented as a geometry-based sensor rather than a pixel-level perception pipeline. This design allows the switching logic and controller interaction to be evaluated in a controlled manner, but it also assumes reliable slot visibility and does not model false positives, false negatives, occlusion, or image-processing latency. Third, the LiDAR feedback used by the TD3 controller is represented by idealized ray-casting range measurements. Sensor noise, dropout, and calibration errors may affect the observation vector and therefore the learned parking policy. Fourth, the TD3 parking controller in this study controls only the steering command, while the longitudinal speed is fixed at 2 m/s during the parking phase. This design simplifies the parking MDP and allows the study to focus on steering-policy learning and terminal docking behavior under a consistent low-speed condition. Therefore, the reported results should be interpreted as steering-policy transfer under a fixed-speed parking setting rather than a fully coupled longitudinal-lateral parking policy. The sensitivity of the learned policy to different parking speeds remains an important limitation and will be examined in future work by introducing speed-varying policies or joint speed-steering control.

These assumptions may lead to optimistic performance compared with a real deployment. Nevertheless, they allow the present study to focus on the feasibility of the hierarchical NMPC-TD3 architecture and the cruise-to-park transition mechanism. Future work will introduce perception uncertainty, LiDAR noise and missing beams, localization drift, actuator delay, dynamic obstacles, mirrored or rotated parking-slot layouts, different aisle widths, more detailed vehicle dynamics, and hardware-in-the-loop or real-vehicle validation to further evaluate the robustness of the proposed framework.

### 4.2. Extension to AI-Defined Vehicle Networks and Multi-Vehicle AVP

Although the present framework focuses on a single ego vehicle, the hierarchical cruise-to-park architecture can be extended to AI-defined vehicle networks and multi-vehicle AVP systems [35]. In a networked parking environment, a higher-level parking management module or infrastructure-side planner could aggregate parking-slot availability, vehicle intents, and local traffic conditions [36]. This network-level layer could assign target slots, resolve conflicts among multiple vehicles, and provide updated cruising references to each ego vehicle.

The NMPC cruising controller in the proposed framework could then track the assigned route under vehicle-level constraints, while the TD3 controller would remain responsible for local low-speed docking after the target slot is confirmed. Vehicle-to-Everything communication could be used to share information such as slot occupancy, intended parking maneuvers, and vehicle priority. For example, a vehicle entering a parking slot could broadcast its intended docking action, while nearby vehicles or the infrastructure could update their cruising targets to avoid local conflicts. In this way, the proposed single-vehicle architecture can serve as a lower-level control and docking module within a broader AI-defined parking network.

### 4.3. Offline Transferability, Network Constraints, and Future Adaptation

The current TD3 parking controller should be interpreted as an offline-trained policy rather than an online adaptive AI-defined vehicle controller. In the reported experiments, the policy is trained in Slot 7 and then deployed to unseen target slots without retraining. Therefore, the results demonstrate intra-lot transferability under a fixed parking-lot layout, but they do not demonstrate continuous adaptation to new parking-lot geometries, new perception conditions, or changing vehicle-network conditions.

In contrast, AI-defined vehicles are expected to improve their models or policies over time through online learning, cloud-edge updates, fleet-level knowledge sharing, or federated learning [37]. Such mechanisms could allow parking policies to adapt to slot shapes, local traffic patterns, and sensor characteristics while reducing the need for complete retraining from scratch. Extending the proposed TD3 module with online fine-tuning, continual learning, or federated policy updates is therefore an important future direction.

Network constraints are another important consideration for extending the proposed framework to AI-defined vehicle networks. The current implementation assumes reliable state estimation, synchronized sensor updates, and stable target-slot information once the free slot is detected and latched. In a networked AVP system, communication delays, packet dropouts, or outdated infrastructure messages could affect slot-availability updates, target assignment, and multi-vehicle coordination [38]. A delayed occupancy update may cause a vehicle to continue toward a slot that has already been assigned to another vehicle, while a delayed coordination message may affect the timing of the cruise-to-park transition.

To address these issues, future work should incorporate uncertainty-aware planning and communication-aware control. For example, the network planner could use conservative estimates of slot availability when communication is intermittent, while the vehicle-level controller could revalidate the target slot before switching to the parking mode. The TD3 parking policy could also be trained with simulated sensing and communication impairments so that it becomes more robust to noisy, delayed, or partially missing observations.

### 4.4. Safety Considerations

Safety guarantees also require further consideration before the proposed framework can be deployed in real-world or multi-vehicle AVP environments. The NMPC cruising controller can explicitly enforce input constraints and can be extended to include additional safety constraints during route tracking. However, the TD3 parking controller is a learned policy and does not by itself provide formal guarantees of collision avoidance or recursive feasibility.

In the present study, safety is encouraged through LiDAR-based observations, invalid-operation penalties, actuator saturation, and closed-loop simulation validation. These mechanisms are useful for training and evaluation, but they are not equivalent to certified safety. For real-world deployment, the learned parking policy should be combined with a supervisory safety layer, such as a control barrier function-based safety filter, a fallback NMPC controller, or an emergency override module that can intervene when predicted collisions or constraint violations are detected [39].

This safety layer would be especially important in networked AVP scenarios, where target-slot changes, delayed coordination messages, and nearby moving vehicles may introduce additional risk during the phase transition and docking process.

## 5. Conclusions

This study presented a hierarchical cruise-to-park automated valet parking framework that combines an NMPC-based controller for the cruising phase with a TD3-based controller for the terminal parking phase in a unified Simulink environment. The objective was to achieve a smooth transition from route following to final parking while maintaining stable control performance throughout the maneuver.

The simulation results show that the proposed framework was able to complete the full parking task under the designed parking-lot setting. During the cruising phase, the NMPC controller kept the vehicle close to the reference path and provided suitable initial conditions for the parking stage. The profiled MPC tracking controller block required an average of 0.0405 s per controller call, which was below the 0.1 s controller sampling time and provided a preliminary indication of computational feasibility in the current MATLAB/Simulink implementation. After the switching condition was satisfied, the TD3 controller completed the terminal maneuver without obvious discontinuity at the transition point. In addition to the training slot, the same controller configuration was validated on six previously unseen target slots within the same parking-lot layout, where successful parking behavior was obtained without retraining. These results indicate that the framework provides a degree of intra-lot target-slot transferability within the tested environment.

The additional baseline comparison showed that PPO achieved faster training convergence, while TD3 provided the most reliable terminal parking performance across the six validation slots. SAC showed competitive behavior in easier cases but became less stable in the most challenging slot. The time-penalty ablation further demonstrated that removing the explicit time penalty can lead to spinning-like or repeated corrective motion near the target region. These results support the use of TD3 with the proposed time-penalized reward formulation as the parking controller in the current cruise-to-park framework.

Although the current study was limited to simulation and a fixed parking-lot layout, it provides a structured basis for further development of end-to-end AVP systems. Future work can extend the framework by introducing speed-varying parking policies, joint longitudinal-lateral control, sensor noise, missing LiDAR beams, localization drift, actuator delay, dynamic obstacles, mirrored or rotated parking layouts, different aisle widths, more diverse parking scenarios, and component-wise reward ablations of the progress reward and steering-smoothness penalty. Further evaluation in higher-fidelity simulation [40,41], hardware-in-the-loop platforms, or real-vehicle experiments [42] will also be necessary before practical deployment. It is also planned to use robust parameter space control design for the parking-space-seeking path tracking in the future [43,44,45,46,47].

## Figures and Tables

**Table 1:** Timing parameters and actuator constraints used in the experiments.

Item	Symbol	Value
Controller sample time	$T_{s}$	0.1 s
Longitudinal speed bound	v	$\left[0,2\right]\;m/s$
Steering angle bound	δ	$\left[-\pi /4,\pi /4\right]\;rad$
Workspace limits	(x,y)	defined by parking-lot map bounds

**Table 2:** NMPC cost weights used in the cruising controller.

Category	Parameter	Value
Tracking	$w_{\mathrm{lat}}$	40
Tracking	$w_{\mathrm{lon}}$	8
Tracking	$w_{\psi }$	25
Effort	$w_{v}$	0.6
Effort	$w_{\delta }$	2.0
Smoothness	$w_{\Delta v}$	6.0
Smoothness	$w_{\Delta \delta }$	10.0
Terminal	$w_{T}$	120

**Table 3:** TD3 training hyperparameters used for parking policy learning.

Hyperparameter	Value	Explanation
Observation dimension $\left(n_{o}\right)$	16	Target-relative pose features and normalized LiDAR ranges
Action dimension $\left(n_{a}\right)$	1	Continuous steering command
Actor hidden layers	[256, 256]	Two fully connected hidden layers
Critic hidden layers	[256, 256]	Two fully connected hidden layers for each critic
Actor learning rate $\left(\alpha_{\pi }\right)$	$3\times 10^{-4}$	Learning rate of the policy network
Critic learning rate $\left(\alpha_{Q}\right)$	$1\times 10^{-3}$	Learning rate of each Q-network
Discount factor (γ)	0.99	Discount factor for future rewards
Experience buffer length $\left(N_{buf}\right)$	$1\times 10^{6}$	Capacity of the replay buffer
Mini-batch size (B)	256	Number of sampled transitions per update
Sample time $\left(T_{s}\right)$	0.1 s	Agent interaction and update interval
Delayed policy update frequency (d)	2	Delayed policy update interval
Target smooth factor (τ)	$5\times 10^{-3}$	Soft target update factor
Target policy smoothing variance $\left({\sigma_{tgt}}^{2}\right)$	0.1	Variance of smoothing noise for target actions
Target policy smoothing bounds	[−0.25, 0.25]	Clipping range for target smoothing noise
Exploration noise standard deviation	0.15	Initial exploration noise level
Exploration noise minimum	0.02	Minimum exploration noise level
Exploration noise decay rate	$2\times 10^{-4}$	Decay rate of exploration noise
Target policy smoothing decay rate	$1\times 10^{-5}$	Decay rate of target smoothing variance
Gradient threshold	1	Gradient clipping threshold
Actor $L_{2}$ regularization	$1\times 10^{-4}$	Regularization for the actor optimizer
Maximum episodes $\left(N_{epi}\right)$	15,000	Training budget
Maximum steps per episode $\left(N_{step}\right)$	500	Episode horizon

**Table 4:** Reward coefficients used for TD3 parking policy learning.

Component	Value	Role
Distance reward scale $\left(c_{dist}\right)$	2	Scales the position-based reward
Longitudinal position weight $\left(w_{x}\right)$	0.05	Penalizes target-relative x-error in distance reward
Lateral position weight $\left(w_{y}\right)$	0.04	Penalizes target-relative y-error in distance reward
Progress reward scale $\left(c_{prog}\right)$	3	Scales the stepwise progress term
Progress saturation lower bound $\left(p_{min}\right)$	0	Prevents negative progress reward
Progress saturation upper bound $\left(p_{max}\right)$	0.1	Limits excessively large progress increments
Orientation reward scale $\left(c_{ori}\right)$	0.1	Scales the heading-alignment reward
Orientation error weight $\left(w_{\psi }\right)$	20	Penalizes heading mismatch in orientation reward
Steering penalty weight $\left(w_{\delta }\right)$	0.05	Penalizes large steering commands
Steering increment penalty weight $\left(w_{\Delta \delta }\right)$	0.1	Penalizes rapid steering variation
Parking bonus $\left(R_{park}\right)$	100	Reward for successful parking
Invalid-operation penalty $\left(R_{invalid}\right)$	−50	Penalty for collision or invalid termination
Time penalty $\left(R_{time}\right)$	−0.02	Per-step penalty during active episode

**Table 5:** Parking performance in validation slots under fixed controller settings.

Slot	Duration (s)	Position Error (m)	Lateral Error (m)	Longitudinal Error (m)	Yaw Error (rad)
14	6.4	0.7403	−0.5601	−0.4841	0.1164
15	6.8	0.6573	−0.3459	−0.5589	0.0362
23	6.5	0.6626	−0.5036	−0.4305	0.0803
39	6.4	0.6655	−0.5504	−0.3740	0.0880
47	7.4	1.3380	1.0361	−0.8466	0.0077
64	6.7	1.1849	−0.7824	−0.8898	0.1623

**Table 6:** Baseline comparison of TD3, PPO, and SAC under the same parking evaluation setup.

Algorithm	Convergence Episode	Successful/Stable Cases	Mean Position Error (m)	Mean Duration (s)	Worst-Case Error (m)
TD3	About 850	6/6	0.8748	6.70	1.338
PPO	About 550	6/6	0.8937	6.75	1.430
SAC	About 650	5/6	1.5055	8.87	5.044

## Data Availability

The original contributions presented in this study are included in the article. Further inquiries can be directed to the corresponding author.
